# Cyanobacteria as a Valuable Natural Resource for Improved Agriculture, Environment, and Plant Protection

**DOI:** 10.1007/s11270-023-06331-7

**Published:** 2023-05-04

**Authors:** Atef M. Abo-Shady, Mohamed El-Anwar H. Osman, Reda M. Gaafar, Gehan A. Ismail, Maysa M. F. El-Nagar

**Affiliations:** Botany Department, Faculty of Science, Tanta University, Tanta, 31527 Egypt

**Keywords:** Abiotic stress, Molecular mechanisms, Plants, Phytohormones, Soils

## Abstract

Taking into consideration, the challenges faced by the environment and agro-ecosystem make increased for suggestions more reliable methods to help increase food security and deal with difficult environmental problems. Environmental factors play a critical role in the growth, development, and productivity of crop plants. Unfavorable changes in these factors, such as abiotic stresses, can result in plant growth deficiencies, yield reductions, long-lasting damage, and even death of the plants. In reflection of this, cyanobacteria are now considered important microorganisms that can improve the fertility of soils and the productivity of crop plants due to their different features like photosynthesis, great biomass yield, ability to fix the atmospheric N_2,_ capability to grow on non-arable lands, and varied water sources. Furthermore, numerous cyanobacteria consist of biologically active substances like pigments, amino acids, polysaccharides, phytohormones, and vitamins that support plant growth enhancement. Many studies have exposed the probable role of these compounds in the alleviation of abiotic stress in crop plants and have concluded with evidence of physiological, biochemical, and molecular mechanisms that confirm that cyanobacteria can decrease the stress and induce plant growth. This review discussed the promising effects of cyanobacteria and their possible mode of action to control the growth and development of crop plants as an effective method to overcome different stresses.

**Graphical Abstract:**

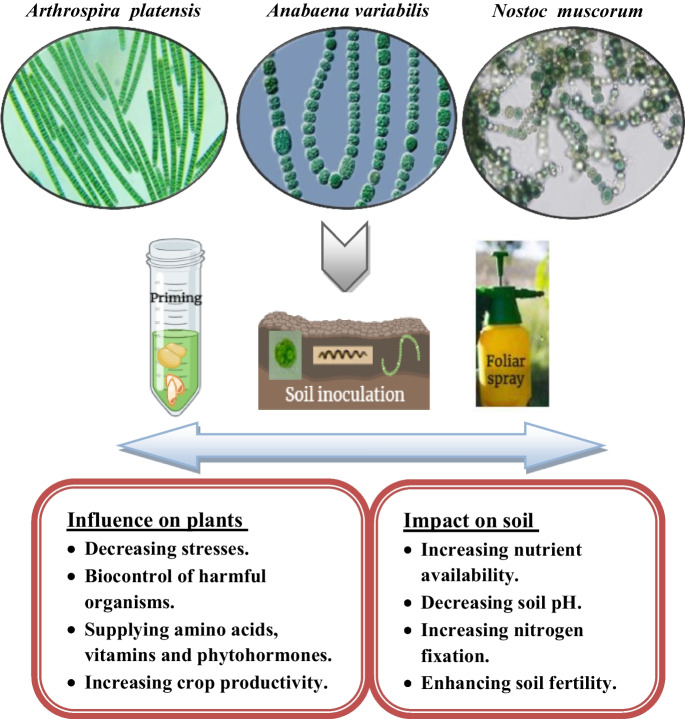

## Introduction

The world exerts continuous efforts to end hunger, food instability, and undernourishment in all its forms in 2030, as reported by the latest 2022 State of Food Security and Nutrition in the World (SOFI) report (FAO et al. 2022(. About 828 million people suffered from hunger globally in 2021 (around 10.5% of the world population) which increased by 46 million in 2020 and 150 million since the COVID-19 pandemic was initiated. Africa has the heftiest local encumbrance. One in five people, or 20.2% of the population in Africa, starved, as compared to 9.1% in Asia, 8.6% in Latin America and the Caribbean, 5.8% in Oceania, and 2.5% in Northern America and Europe together (FAO et al. 2022(. Also, the continuous increase in the worldwide population caused an augmented request for different food supplies (El-shenody et al. 2023; Pathak et al. 2018; Ronga et al. 2019), besides, the reduction in available area for food crop production, infrequent water resources, accretion of xenobiotic compounds in the soils; and deficient soil quality. So, the main issues facing hunger are agricultural maintenance challenges, environmental subsidies, and climate change.

The present agricultural applications are deeply reliant on the application of synthetic chemicals such as fertilizers and pesticides, both as plant growth stimulators and as an agent for protecting plants from different stress conditions, and that facilitated many developing countries to meet the food necessity of their people (Ashour et al. 2021, 2023; Dmytryk and Chojnacka, 2018; Hassan et al. 2021).

Unfortunately, The repetition of using chemicals in agriculture may accumulate in the plants and soil, releasing and forming environmentally harmful products that can be a danger to humans (Pan et al. 2019). For example, about 50% of the useful nitrogen fertilizer is essentially used by plants, and the remaining 50% causes damage in surface waters (Collos and Harrison, 2014). So, it is a big challenge to available the food requirements of the population with limited resources and without worsening the environmental quality (Singh and Strong, 2016).

Cyanobacteria are an ideal solution for this problem due to their environment-friendly and low-cost farming. Since thousands of years ago, cyanobacterial biomass has been widely used in agriculture, but in the twentieth century, increased beneficial products obtained from cyanobacterial extracts have attracted the attention of farmers worldwide (Kumar et al. 2022; Pathak et al. 2018). As a result, researchers have focused their efforts on biologically-based products such as cyanobacteria, which have been evaluated as crop protection agents as well as for their bio-stimulating potential (Górka et al. 2018).

Cyanobacteria are prokaryotic microorganisms, and some are heterotrophs, capable of using various sources of carbon and organic nitrogen (Pham et al. 2017). It can be tailored to many environmental changes due to its diversity in morphological characteristics (Singh et al. 2014). An additional feature that makes algae and cyanobacteria more appropriate is that they do not need arable land for their growth. Cyanobacteria can be alive in the desert, in contaminated and stressed soils, and in other excessive environmental conditions (Rossi et al. 2017). Furthermore, they could grow with high productivity on remaining nutrients while supplementing yields of lipids (20–65% of dry weight), proteins, total fibers (33–50% higher than plants), and carbohydrates (El Shafay et al. 2021; Guihéneuf et al. 2016).

Furthermore, it can produce several bioactive compounds that can stimulate crop growth/protect and improve the soil nutrient status. Cyanobacteria are also beneficial for wastewater management and have the capability to break down numerous toxic compounds even pesticides (Cohen, 2006).

This review focused on providing an overview of cyanobacterial roles in the improvement of plant growth and yield depending on their biostimulants. In addition, it explains their roles in the alleviation of abiotic stress conditions on crop plants.

## Cyanobacteria Growth and Extraction

Cyanobacteria grow earlier than plants and are characterized by a simple genetic system and a high yield of biomass and metabolites (Wijffels et al. 2013). Good results for their products are dependent on the strain chosen and the effectiveness of the cultivation system (Balasubramaniam et al. 2021). Several basic parameters adjust the growth of the strains, including (1) light intensity, (2) pH, (3) gas exchange, (4) nutrient source, and (5) light/dark cycling (Balasubramaniam et al. 2021).

**Fig. 1:**
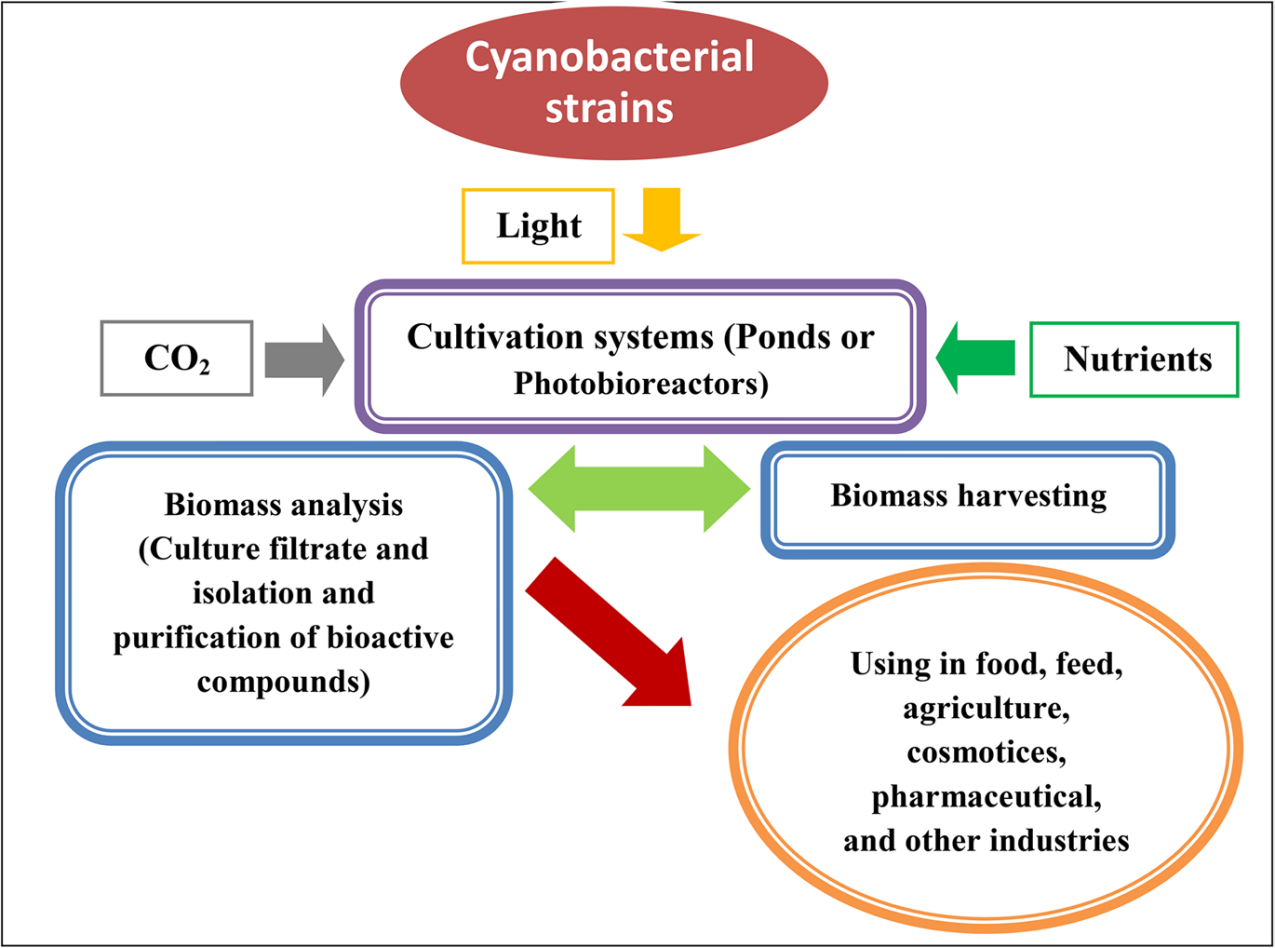
Cyanobacterial strains’ culture, harvesting, and downstream use

Cyanobacteria mass production might be achieved by two basic systems: open reservoirs (ponds or tanks), or closed containers (photobioreactors), which are supported by natural or artificial illumination (Fig. 1).

## Biostimulants (Biomolecules) from Cyanobacteria

The possible applications of cyanobacteria embrace agriculture, wastewater management, bioenergy, and bioactive value-added compounds (Odjadjare et al. 2017). According to the literature, cyanobacterial metabolites can play an important role in soil decontamination and fertilization, plant protection against biotic and abiotic stress factors, and plant development (Górka et al. 2018; Ronga et al. 2019). Besides their role in adding organic nitrogen and complementary mineral nutrition to the soil, they are known as sources of numerous biologically active compounds and secondary metabolites including phytohormones (auxins, gibberellins, and cytokinins) that can be used in biotechnological and industrial fields (Guihéneuf et al. 2016). These bioactive substances can affect plant gene expression and encourage the accumulation of a wide range of compounds that help in the stimulation of plant growth and protection against biotic and abiotic stress (Han et al. 2018; Pan et al. 2019) (Table 1). Cyanobacteria are generally gaining attention as plant-growth-promoting and biocontrol agents in diverse crops, including rice, wheat, cotton, and legumes (Prasanna et al. 2015). The inoculation of these organisms influences various metabolic processes in plants since they activate the production of defense proteins and enzymes that lead to a greater immunity of plants against pathogens (Gonçalves, 2021). It is known that the inoculation of cyanobacteria directly in the soil or by seed engagement or priming causes an increase in the germination rate, better development of plants, and a higher production yield in a wide variety of cereal, horticultural, and vegetable crops (Singh et al. 2017; Toribio et al. 2021). From what was mentioned above, cyanobacteria are considered platforms for the potential development of products for soil improvement and crop production and protection, such as biofertilizers, organic fertilizers, biostimulants, and biocontrol agents as summarized in Table 2.

**Table 1:** Cyanobacterial strains metabolites (Kollmen and Strieth, 2022)

Class	Metabolites	Cyanobacterial strains
Phytohormones	Auxins, abscisic acid, cytokinins, gibberellins, ethylene	*Anabaena* sp., *Nostoc* sp., *Oscillatori*a sp., *Phormidium* sp., *Rhodospirillum* sp., *Scytonem*a sp., *Synechocystis* sp., and *Westiellopsis prolifica*
Phenolic compounds	Flavonoids, phenolic acids, cell wall phenolics	*Anabaena* sp., *Arthrospira* sp., *Calothrix*, *Chroococcidiopsis,* *Leptolyngbya*, *Nostoc* sp., *Oscillatoria, Phormidium*
Terpenoids	Isoprene, limonene, β-phellandrene, linalool, farnesene, bisabole	*Anabaena* ap., *Synechocystis* sp., *Synechococcus* sp.
Carotenoids	β-carotene, astaxanthin, canthaxanthin, zeaxanthin, lutein, lycopene, phytoene, echinenone	*Anabaena* sp., *Cylindrospermum* sp., *Microcystis* sp., *Nostoc* sp., *Oscillatoria* sp., *Phormidium* sp., *Synechococcus* sp., *Spirulina* sp., *Tolypothrix* sp.
Peptides	Peptides, a free amino acids, proteins	*Aphanizomenon flos-aquae*, *Calothrix ghosei*, *Cylindrospermum musciola*, *Hapalosiphon intricatus*, *Microcystis aeruginosa*, *Nostoc* *muscorum*, *Nostoc* sp.,
Polysaccharides	β-glucans, chitin, lipopolysaccharides, carrageenan	*Arthrospira platensis*, *Nostoc* *muscorum*, *Cylindrospermum musciola*
Vitamins	Riboflavin, ascorbic acid, thiamine, cobalamine, pyridoxine, nicotinic acid, folic acid, phenothene	*Anabaena* sp., *Chroococcus* *mimulus, Microcystis pulverana*, *Nostoc* sp., *Nostoc muscorum*, *Oscillatoria jasorvensis*, *Phormidium bijugatum*, *Arthrospira*

**Table 2:** Cyanobacterial biomass and/or extracts’ main known activities in crop plants (Gonçalves, 2021)

Type	Mode of action	Influence on crops
Biostimulants	Plants	Protection and production
Biofertilizers	Soils	Nutrition
Biopesticides	Pathogenic organisms	Protection

Taking into account their potential benefits for the development of workable agriculture, both biomass, and extracts from cyanobacteria are commercially available on the market (Górka et al. 2018), as abridged in Table 3.

**Table 3:** Some common cyanobacterial biostimulants are currently on the market (Gonçalves, 2021)

Commercial name	Species	Mode of application
Spiragro Spiragrow	*Arthrospira platensis*	Foliar and radical
Floralgal Algafert	*Arthrospira* sp*.*	Foliar and radical
Shwe Awzar	*Arthrospira* sp*.*	Radical-soil conditioner
Microp	Unspecified cyanobacteria	Radical-soil conditioner
Agrialgae Phycoterra	Unspecified cyanobacteria	Foliar and radical

**Fig. 2:**
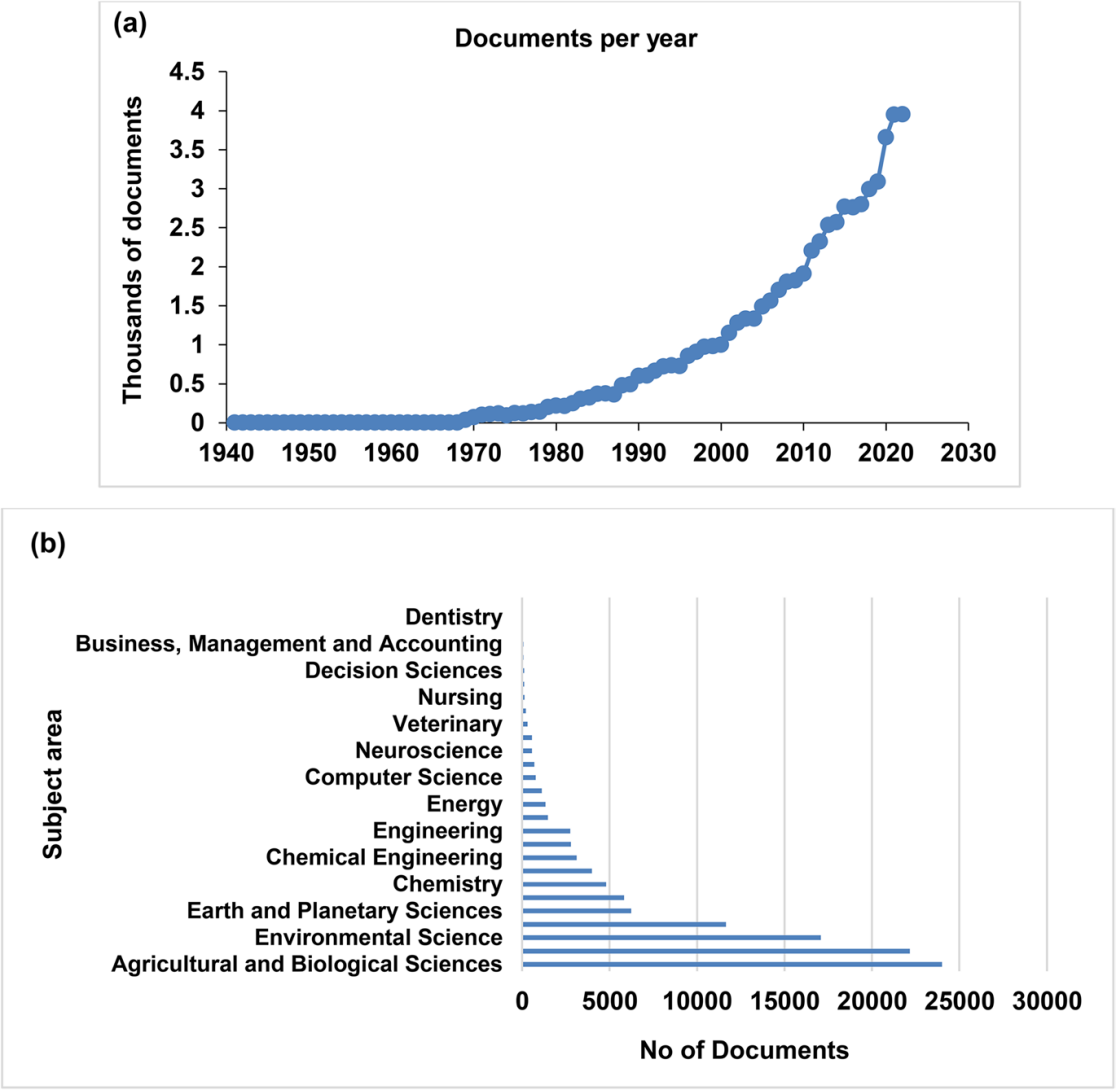
Cyanobacteria by year (**a**) and by subject area (**b**) appraised in the Scopus® database until 2022 (updated to February 2023)

The attention to cyanobacteria is growing, as revealed by several studies in many fields (Fig. 2a) like Biological Sciences, Agricultural, Biochemistry, Environmental Science, Genetics and Molecular Biology, Immunology, and Microbiology (Fig. 2b).

## Role of Cyanobacteria in Agriculture

### Plant Growth and Yield Improvement

Cyanobacteria can be applied to monocot and dicot crops as biofertilizers to raise plant growth and crop yield. Cyanobacteria as biofertilizers are inexpensive, they cost one-third of the price of chemical fertilizers (Prasanna et al. 2013). *Nostoc* and *Anabaena* are familiar genera for plant growth promotion (Prasanna et al. 2016). They can supply nitrogen to the plant by using nitrogenases enzyme to fix atmospheric nitrogen (Meeks and Elhai, 2002).

This helps plants to grow without nitrogen-deficient soils and eliminates the need to use expensive nitrogen fertilizers, which are frequently a source of pollution (Li et al. 2019). In the same context, Osman et al. (2020) reported that treatment of broad bean seeds with biomass of *Nostoc muscorum* induced growth parameters of *root length (30%), shoot length (44%), root fresh weight (2-fold), shoot fresh weight (1.5-fold), root dry weight (67%), and shoot dry weight* (1.6-fold). Photosynthetic pigment, carbohydrate, and protein contents were also increased by 52%, 20%, and 1.7-fold, respectively. *Moreover,* algal treatments improved the activity of antioxidant enzymes (peroxidase and catalase) and were accompanied by a decline in lipid peroxidation (Osman et al. 2020). In another study, *Spirulina platensis* was applied to radish (*Raphanus sativus*) with its filtrate as seed soaking and foliar spray in addition to its homogenate as a seed coating treatment. *Spirulina* increased growth, chlorophyll, and element content as compared to the control of radish seedlings (Godlewska et al. 2019). Crude bio-extracts obtained from 18 cyanobacteria and microalgae species were recommended as biostimulants for plant growth, nutrient uptake, chlorophyll content, and metabolite profiles of the tomato plant (Mutale-Joan et al. 2020). The authors detected that root and shoot lengths of the plant were significantly developed by 112.65% and 53.70% in response to *Aphanothece* sp treatment, respectively. In the meantime, the uptake of nitrogen, phosphorus, and potassium was amplified by 185.17%, 119.36%, and 78.04%, respectively, compared with the control plants. In the same manner, Osman et al. (2021) reported that priming wheat grains in *Arthrospira platensis* and *Nostoc muscorum* aqueous extract caused wheat growth stimulation and improvement in various morphological growth parameters. In addition, the biochemical content of pigments, carbohydrates, and proteins was boosted in the wheat seedlings. Moreover, using *Anabaena laxa* and *Anabaena rhizobium* caused an increase in yield by 104% and the nitrogen content by 50% in chickpea (*Cicer arietinum L*.) plants as compared to control plants (Bidyarani et al. 2016). In another experiment, *Anabaena laxa* amplified the yield by up to 39% and the protein content by 11% for pea plants (Prasanna et al. 2017).

In recent years, cyanobacterial polysaccharides have been applied to plants and stimulants for their properties and signaling capabilities (Elarroussi et al. 2016; Farid et al. 2019). *A. platensis* crude polysaccharides extract was applied to tomatoes and peppers by foliar spraying. The treatments amplified shoot dry weight by 1.4-fold in both species of plants, while the positive effects on root weight were much more noticeable in tomatoes (2.30-fold) than in peppers (67%) (Elarroussi et al. 2016). Crude polysaccharide extracts can hold other bioactive metabolites that may contribute to the experimental effects. For example, crude polysaccharides extracted from *Phormidium tenue*, consisting of 58% carbohydrates and 15% proteins have been shown to induce growth and superoxide dismutase activity in seedlings of the shrub *Caragana korshinskii* (Xu et al. 2013). Besides, it has been revealed that crude polysaccharides extracted from *A. platensis* contain phenolic compounds (about 45 mg gallic acid equivalent to g^−1^ of biomass), which show antioxidant activities in plants (Chaiklahan et al. 2013). Together, these findings support the hypothesis that cyanobacterial polysaccharides might be an active source of plant biostimulants for crop enhancement and protection against abiotic stresses (Elarroussi et al. 2016). Also, several metabolic pathways, such as photosynthesis and nitrate assimilation, seem to be affected by treatments with cyanobacterial polysaccharides. A gas chromatography–mass spectrometry (GC-MS) metabolomic examination also showed an increase in phytosterols (Farid et al. 2019). The rise in plant sterols might lead to the production of brassinosteroids, a group of oxidized steroids with hormonal activities accountable for increasing the effectiveness of photosynthetic carbon fixation and avoiding damage to photosynthetic pigments through stresses (Siddiqui et al. 2018). Table 4 listed some studies that exposed the beneficial roles of using cyanobacterial species in the growing of economic crops.

**Table 4:** The beneficial roles of different cyanobacterial strains on plants

Cyanobacterial strains	Plant	Beneficial role	Reference
*Aphanothece* sp	Tomato	Amplified uptake of nitrogen, phosphorus, and potassium by 185.17%, 119.36%, and 78.04%, respectively	(Mutale-Joan et al. 2020)
*Anabaena laxa*	Chickpea	50 % higher grains yield	(Bidyarani et al. 2015)
*Anabaena* sp., *Anabaena doliolum*, *Nostoc carneum* and *Nostoc piscinale*	Maize	Yields were increased with 20–30% increases in all inoculated treatments	(Prasanna et al. 2016)
*Nostoc muscorum*	Barley	Increased in proteins (45.95%), amino acids (39.13), and nutrient content [N (28.4%), K (24.3%), Ca (12.9%), Mg (29.06%), and Fe (13.8%)	(Abo-Shady et al. 2018)
*Nostoc muscorum*	Broad bean	Induced growth parameters of *root length (30%), shoot length (44%), root fresh weight (2-fold), shoot fresh weight (1.5-fold), root dry weight (67%), and shoot dry weight* (1.6-fold)	(Osman et al. 2020)
*Aphanothece* sp	Tomato	Root and shoot lengths of the plant were significantly developed by 112.65% and 53.70%, respectively	(Mutale-Joan et al. 2020)
*Arthrospira Platensis*	Tomatoes and peppers	Induced shoot dry weight by 1.4-fold in both species of plants, while the positive effects on root weight were much more in tomatoes (2.30-fold) than in peppers (67%)	(Elarroussi et al. 2016)
*Anabaena laxa*	Pea	Increased the yield by up to 39% and the protein content by 11%	(Prasanna et al. 2017)

### Cyanobacteria as Phytoremediation Tool

Agricultural practices have influenced the environment's air, water, and soil. These environmental factors depend on factors like location and management practices used, such as fertilizers application. Also, adding nutrients and pesticides can cause runoff from agricultural fields into surface water and groundwater (Abdelsalam et al. 2019; Rohila et al. 2017). Biological treatment with cyanobacteria to remove pollutants is called (Bioremediation). Cyanobacteria could be used for the bioremediation of numerous organic pollutants like heavy metals, pesticides, surfactants, phenol, and catechol (Kumar et al. 2016; Singh et al. 2016) using several ways. Their capability to remove pollutants results from their high photosynthetic activity, which offers huge biomasses. These biomasses consist of numerous bioactive substances that could be used in many useful applications (Gonçalves, 2021). Furthermore, many species of cyanobacteria can grow best in contaminated environments, such as *Oscillatoria limosa*, *Oscillatoria tenuis*, *Oscillatoria princeps*, *Anabaena torulosa, Nostoc* sp*.,* and *Phormidium uncinatum* (Gonçalves, 2021). Likewise, *Anabaena flosaquae* had the highest absorption ability for pollutants as compared to *Microcystis aeruginosa* (Corpuz et al. 2021).

#### Methods of Bioremediation

Cyanobacteria can accumulate organic or inorganic toxic substances and produce several detoxifying mechanisms, like bioaccumulation, biosorption, biotransformation, and biodegradation (Mondal et al. 2019). Biosorption is a physicochemical method that depends on different factors such as absorption, adsorption, surface area, ion exchange, and precipitation. Biosorption is a perfect method for the removal of many pollutants (phenolic compounds, heavy metals, herbicides, and pesticides). Moreover, it is friendly to the environment and a cheaper method for removing pollutants (Gadd, 2014). Cyanobacterial biodegradation happens extracellularly, intracellularly, or both, where the primary degradation arises extracellularly (Ventura et al. 2017). The amount of removal and biodegradation of pollutants are influenced by their concentration, cyanobacterial biomass, species, growth phase, and environmental conditions (Abou-El-Souod and El-Sheekh, 2016).

#### Heavy Metals and Organic Pollutant Removal

Heavy metals are certainly found in the environment at actual low levels, but due to human activities and industrial waste their concentration in the environment has extremely amplified. Heavy metals are very dangerous toxicants for living organisms thus they can bind to some cellular components, counting enzymes, proteins, and nucleic acids, and upset their normal functions (Abdelsalam et al. 2019; Das et al. 2008). Cyanobacteria can remove numerous heavy metals such as cadmium (Cd), chromium (Cr), copper (Cu), lead (Pb), and zinc (Zn) (Das et al. 2008) (Table 5).

**Table 5:** Heavy metal, organic pollutant, and pesticides removal via some species of Cyanobacteria

Heavy metals	Cyanobacteria	Removal percent	References
Cd	*Nostoc linckia* and *Nostoc rivularis*	2–10-fold	(El-Enany and Issa, 2000)
Zn	*Nostoc linckia* and *Nostoc rivularis*	10–30-fold	(El-Enany and Issa, 2000)
Cu, Co, Pb, and Mn	*Nostoc muscorum and Anabaena subcylindrica*	12.5–81.8, 11.8–33.7, 26.4–100, and 32.7–100%, respectively	(El-Sheekh et al. 2005)
Zn	*Spirulina platensis*	87%, 80%, and 70.5% when its initial concentration was 0.5, 1, and 2 mg/L, respectively	(Meng et al. 2012)
Cr	*Nostoc* PCC7936	50%	(Colica et al. 2010)
Sr	*Gloeomargarita lithophora* and *Cyanothece* sp	–	(Cam et al. 2016)
Organic pollutants	Cyanobacteria		References
Phenol	*Ochromonas danica*	65%	(Semple and Cain, 1996)
Diesel 99.5% (0.6% v/v)	*Phormidium* sp., *Oscillatoria* sp., and *Chroococcus* sp.	94%	(Chavan and Mukherji, 2008)
Total petroleum hydrocarbon 99% (diesel 0.6% v/v)	*Phormidium* sp., *Oscillatoria* sp., and *Chroococcus* sp.	99%	(Chavan and Mukherji, 2010)
Phenanthrene	*Selenastrum capricornutum* and *Microcystis aeruginosa*	96–100%	(Chan et al. 2006 and Bai et al. 2016)
Dimethyl phthalate	*Synechocystis* sp. PCC6803 *Synechococcus* sp. PCC7942, and *Cyanothece* sp. PCC7822	11.8%	(Zhang et al. 2016)
Pesticides	Cyanobacteria		References
Glyphosate (H)	*Anabaena* sp*.,* and *Nostoc* sp	–	(Forlani et al. 2008)
Chlorpyrifos (I)	*Spirulina platensis* and *Spirulina maxima*	60%	
Carbofuran (I)	*Nostoc hatei*	12%	(Jha and Mishra, 2005)
Fluroxypyr (H)	*Chlamydomonas reinhardtii*	57%	(Zhang et al. 2011)
Mancozeb (F)	*Nostoc ellipsosporum,* and *Tolypothrix tenuis*	–	(Barton et al. 2004)

Heavy metals like Cd in soils can be prevented from translocation from roots to shoots as a response to seed priming in *Spirulina platensis* and can also stimulate seed germination and improve maize plant growth (Seifikalhor et al. 2020). Additionally, *Limnococcus* sp., *Nostoc muscorum*, and *Synechococcus* sp*.* can remove a wide variety of heavy metal ions like Cu, Ni, Pb, Cd, Zn, and Co (Al-Amin et al. 2021). In the same manner, Cyanobacterial can remove different organic compounds from different systems (Table 5). Likewise, *Lyngby lagerlerimi*, *Nostoc linkia*, and *Oleria rubescens* have been widely used to remove phenolic pollutants (El-Sheekh et al. 2012). Also, *Spirulina maxima* can degrade phenolic compounds, which are reflected as very toxic pollutants in the USA (Ebele et al. 2017).

#### Pesticides and Herbicides Removal

Pesticides contaminated soils and waters due to their bio-accumulative and persistent nature (Mastovska and Wylie, 2012). Their presence is harmful to plants, ecosystems, drinking water, and human health (Mastovska and Wylie, 2012). Pesticides are one of the most vital agricultural involvements that affect the quality and production of crop plants. It can be classified into herbicides (weed killers), fungicides (fungal destroyers), nematicides (nematode killers), insecticides (insect killers), and rodenticides (vertebrate poisons) (de Souza et al. 2020). In contrast, their excessive use and toxic properties make them a major issue for public health and the environment (Nicolopoulou-Stamati et al. 2016). Moreover, pesticides, accidental or highly occupational, result in many side effects such as dermatological, neurological, carcinogenic, respiratory, and reproductive, which lead finally to hospitalization and death (Thakur et al. 2014). An earlier study that reflects the beneficial role of cyanobacteria as *Anabaena* sp., *Nostoc* sp., and *Arthrospira* sp. can use glyphosate herbicides as a source of phosphorus, which helps in the removal of this herbicide from polluted soil (Forlani et al. 2008). Several cyanobacterial species were used to bioremediate toxic pesticides Table 5. Also, it has been found that *Synechocystis* sp. and *Phormidium* sp. can bioabsorb and remove the insecticide imidacloprid from the soil (Aminfarzaneh and Duygu, 2010). In the same way, *Scytonema hofmanni* and *Fischerella* sp. can remove the insecticide methyl parathion (Tiwari et al. 2017). In herbicide situations, priming faba bean seeds in *Spirulina platensis* stimulates the production of some amino acids that can protect them from the adverse effects of the fusillade herbicide on the plants (Osman et al. 2016). Besides, *Microcystis aeruginosa* was found to break down phenyl urea herbicides (Bayazıt et al. 2020). Moreover, *Spirulina* sp., *Westiellopsis* sp., and *Oscillatoria* sp. are recorded as the most generally used cyanobacteria for wastewater treatment (Das et al. 2017).

*I* insecticide, *F* fungicide, *H* herbicide

### Cyanobacteria Protection Against Plant Abiotic Stresses

#### Effect of Different Abiotic Stresses on Crop Plants

**Fig. 3:**
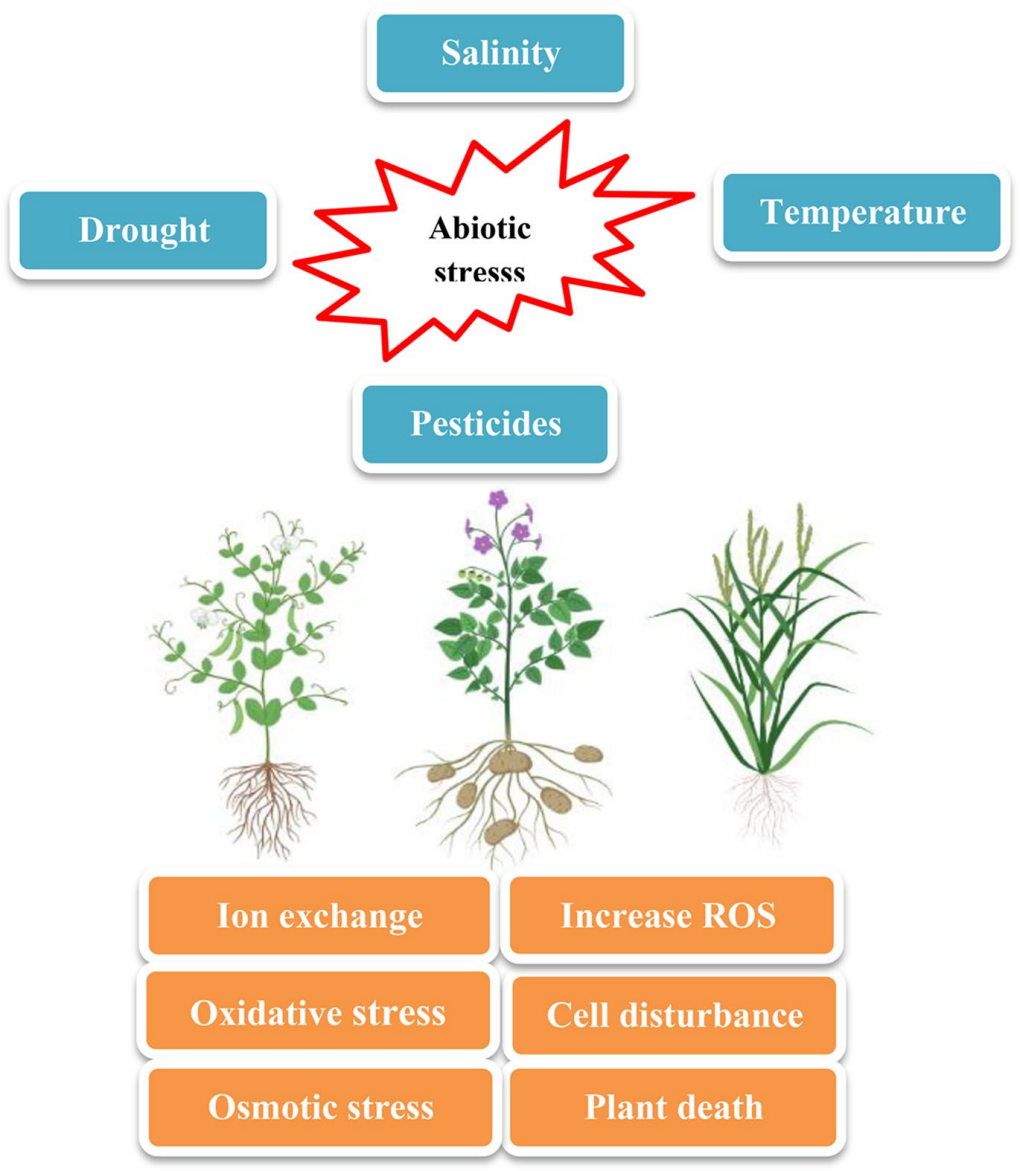
Effect of abiotic stress on crop plants

Plant stress can be classified into several types by numerous factors, including the type of stress (biotic and abiotic), the effect of the stress, and the persistence of the stress (short-term and long-term stresses) (Kranner et al. 2010). Alternatively, plant stresses can be classified into internal stresses (that come from within the plant) and external stresses (that exist outside the plant). When one or more stresses change the optimal conditions of the plant, it uses a special mechanism called “stress sensing” to detect this variation. When one or more stresses change the optimal conditions of the plant, it uses a special mechanism called “stress sensing” to detect this variation. There are four main phases: (1) alarm, (2) resistance, (3) exhaustion, and (4) regeneration phases of plant stress sensing and response based on the duration and intensity of the stress (Kranner et al. 2010). Stress resistance depends on species, genotype, age of the plant, tissue identity, duration, severity, and rate of stress. Many abiotic stresses such as salinity, temperature, drought, and pesticides (ex: herbicides) are established in plants as osmotic stresses, leading to the accumulation of reactive oxygen species (ROS) that harm carbohydrates, proteins, lipids, DNA, and also cause abnormal cell signaling (Mala et al. 2017) (Fig. 3). Abiotic stresses result in about half of all yield losses. For example, high temperatures (20%), low temperatures (7%), salinity (10%), drought (9%), and all other abiotic stresses account for about 4% (Ningombam et al. 2021).

##### Cold and Heat Stress

Cold and heat stress are preventive factors for stimulating crops (Ruelland et al. 2009). Low-temperature stress disturbs plant reproductive stages, causing late flowering and pollen production which causes crop yield reduction (Yadav, 2010) (Table 6). Besides that, cold stress-stimulated membrane dehydration (Saito and Matsuda, 2010). Likewise, *Jatropha curcas* is a key bioenergy crop, but it cannot yield biofuel under cold stress (Wang et al. 2018). Low-temperature stress not only declines grain yield but also upsets crop grain value. Also, Atayee and Noori (2020) observed that cold stress induced the ability to injury and death of many plants through a variety of physiological disruptions. As reported by Joshi et al. (2016), not only low temperatures but also high temperatures caused losses in grain weight and injury during grain developmental stages. In the same manner, the biosynthesis of phenolics, proline, secondary metabolites, and grain growth was inhabited as a result of heat stress (Kamal et al. 2017).

**Table 6:** Effect of different abiotic stresses on crop plants

Crop plants and stress	Effects	Reduction percent	References
Low temperatures Wheat Barley Canola Chickpea Field pea	Decrease grains yield and quality	–	
High temperatures Wheat	losses in grain weight and injury during grain developmental stages	Tiller number (70%), grain yield (67.3%), 1000-grain weight (47%), and vigour Index II (52.7%)	(Joshi et al. 2016)
High temperatures Cotton	Biosynthesis of phenolics, proline, secondary metabolites, and grain growth was inhabited	–	(Kamal et al. 2017)
Salinity Maize	Decreased in germination rate, root length, and shoot length	32%, 80%, and 78% in germination rate, root length, and shoot length, respectively,	Khodarahmpour et al. (2011)
Drought *Isatis indigotica*Fort	Decreased chlorophyll content	31%	(Hao et al. 2013)
Drought Legumes grain	Destroys many stages of development, especially the generation and function of reproductive organs	27 to 87%	(Farooq et al. 2017)
Drought *Ornamental shrubs*	Reduction in plant physiological, metabolic, enzymatic, and grain yield processes	50%	(Toscano et al. 2016)
Glyphosate herbicide *Dimorphandra wilsonii*	Simulated ROS that leads to a reduction in the germination of seeds by dropping the rates of seed respiration	–	(Gomes et al. 2017)
Roundup herbicide Maize	Setback the seed germination process	–	(Gomes et al. 2019)

##### Salinity and Drought Stress

Salinity is one of the most abiotic stresses that affect plant growth and productivity (Table 6). Khodarahmpour et al. (2011) reported that in maize crops there was a reduction of 32%, 80%, and 78% in germination rate, root length, and shoot length, respectively, as a response to salinity stress. Panuccio et al. (2014) noted that shoot length and root length were significantly reduced in the response to the salinity stress. In the same manner, increased salinity had side effects on sorghum production and yield components (Shakeri et al. 2017). Drought stress can happen at any stage of plant production. Normal plant growth phases are very sensitive to soil moisture, which significantly caused a decline in crop yield (Krishnamurthy et al. 2010). Hao et al. (2013) noted that drought-stressed plants decreased chlorophyll content by 31% as compared to control plants. Also, Farooq et al. (2017) observed that drought destroys many stages of development, especially the generation and function of reproductive organs, by 27 to 87%. Moreover, drought stress caused a 50% reduction in plant physiological, metabolic, enzymatic, and grain yield processes (Toscano et al. 2016).

##### Herbicides Stress

Although herbicides can help in increasing crops' yield, not all herbicides can get to their targets, and an extensive part of them can remain in soils or be absorbed by other non-target plants (Parween et al. 2016). Repetitive use of one or more herbicides with the same mode of action can result in the generation of resistance in the weed population (Sharma et al. 2019). For example, Gomes et al. )^2017^) reported that Glyphosate simulated ROS in *Dimorphandra wilsonii* leads to a reduction in the germination of seeds by dropping the rates of seed respiration. Another formulation of glyphosate herbicides (Roundup) may setback the seed germination process of maize (Gomes et al. 2019). Similarly, Subedi et al. (2017) experimented with glyphosate herbicide on *Lens culinaris L.* and they established that seed germination declined as compared to the control. In the same context, Wang et al. (2018) reported that the application of herbicides can affect plant growth and progress and result in yield reduction. Herbicides stimulate reactive oxygen species (ROS) as a secondary effect when applied to plant cells (Caverzan et al. 2019), which react to different cell consistent (like proteins, lipids, pigments, and nucleic acids) and finally trigger lipid peroxidation, membrane damage, and enzyme activity disturbance (Singh et al. 2017) (Table 6).

#### Plants Detoxification Defense System

**Fig. 4:**
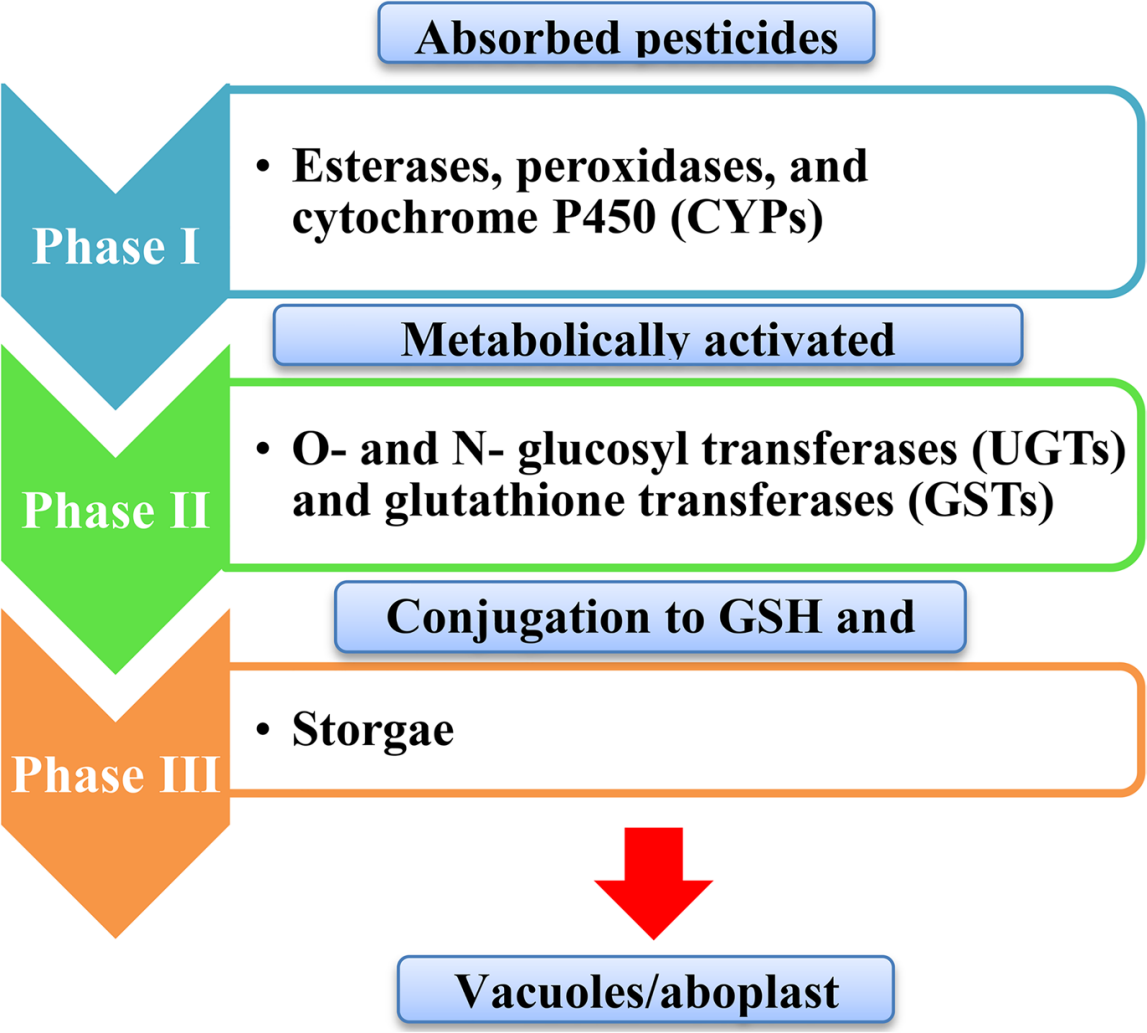
Flow chart of the three-step pesticide detoxification system

Plants have adopted a biotransformation process or detoxification system of xenobiotics (a chemical substance found within an organism that is not naturally produced or expected to be present within the organism) to protect themselves from the destructive attack of such compounds. These detoxification systems can be subdivided into four phases. The 1st phase of xenobiotics metabolism is manipulated by esterases, peroxidases, and cytochrome P450-dependent monooxygenases (CYPs), which are present in many isoforms (Coleman et al. 1997). The most common CYPs reactions are hydroxylation (Kreuz et al. 1996). Products from phase I do not always result in decreased phytotoxicity. Some xenobiotics already involve functional groups (OH, NH_2_, and COOH) and have exceeded the second phase completely. The 2nd phase of metabolism is stimulated by conjugating hydrophilic enzymes like transferases (O– and N–glucosyl transferases (UGTs) and glutathione transferases (GSTs) to the xenobiotic metabolite to form a water-soluble conjugate (Gaillard et al. 1994). The 3rd phase of metabolism comprises xenobiotic conjugates being placed in the large central vacuole of the cell, remarkably with the aid of adenosine triphosphate binding cassette transporter proteins (ABC) (Edwards et al. 2005) (Fig. 4). In the 4th phase of xenobiotic metabolism, products formed were introduced into the vacuole and could be transferred out into the cytoplasm and combined into cell wall components or other macromolecules (Gaillard et al. 1994; Edwards et al. 2005). Of such systems (phases), the one on which many recent studies have focused is the detoxification of herbicides through conjugation to the glutathione transferases (GSTs) (Baek et al. 2019; Sun et al. 2017; Taylor et al. 2013). The inhibitory outcome of abiotic stresses on plant growth is shown at many levels and includes a varied range of cellular processes that are controlled by hormones, amino acids, polyamines, polysaccharides, and enzymes that might be changed during stress (Wally et al. 2013).

#### Cyanobacteria’s Role Against Plant Abiotic Stresses

The decrease of the harmful effect of abiotic stresses observed in plants due to the interaction with cyanobacteria, which has been proved directly, by its action in the soil, or indirectly, due to the activation of specific responses in plants (Singh, 2014)

In the same context, *A. platensis* aqueous extract has been shown to contain a high amount of phytohormones like jasmonic acid, abscisic acid, and cytokinins, which participate in plant response to abiotic stresses (Tejada-Ruiz et al. 2020). Interestingly, the presence of salicylic acid, a vital signaling molecule responsible for the stimulation of defense responses in plants, was identified in the extracts of 28 cyanobacterial strains (Toribio et al. 2020). l-amino acids can work as signaling molecules to alleviate the damage caused by abiotic stresses (Oosten et al. 2017). New reports show that melatonin, a derivative of L-tryptophan, can help major seeds tolerate opposing environmental conditions at the germination stage (Kołodziejczyk et al. 2016). *A. platensis* biomass is known to be rich in l-amino acids and has been reported to motivate carbon metabolism, chlorophyll synthesis, and sugar content (Mógor et al. 2018), likewise providing useful properties through stress. Other cyanobacteria (*Synechocystis* sp*.* and *Anabaena* sp.) have been revealed to accumulate polyamines under stressful conditions, which synthesis comes from the decarboxylation of l-amino acids like l-arginine and l-ornithine (Mógor et al. 2017).

Application of cyanobacteria in crop plant fields alleviates adverse effects caused by salinity stress. Shariatmadari et al. (2015) observed that the application of cyanobacteria (*Anabaena vaginicola* ISB42, *Anabaena oscillarioides* ISB46, *Anabaena torulosa*, *Anabaena sph*aerica ISB23, *Trichormus ellipsosporus*, and *Nostoc calcicola*) in Mentha piperita fields exposed to salinity stress stimulated plant growth and oil content. Likewise, Rady et al. (2018) found that cyanobacteria alone or in combination with glutathione and ascorbic acid inoculated in salt-stressed soil cultivated with bean plants improved growth parameters like plant length, number of leaves, and fresh and dry weights of plants, as well as yield parameters. Moreover, photosynthetic pigments, relative water, stability of membrane, carbohydrates, proline, ascorbic acid, glutathione, N, P, and K+ion content, superoxide dismutase, and catalase activities were also stimulated compared to control plants. Furthermore, Brito et al. (2022) found that the cyanobacterial species *Oculatella lusitanica* LEGE stimulated salinity stress resistance in lettuce plants by increasing the non-enzymatic antioxidant system (H_2_O_2_, proline, and reduced glutathione). Regarding drought cases, soils inoculated by *Spirulina meneghiniana* and *Anabaena oryzae* improved lettuce plants’ growth as compared with non-inoculated soil plants (Ibraheem, 2007). Similarly, priming seeds of *Senna notabilis* and *Acacia hilliana* before cultivation in *Microcoleus* sp. and *Nostoc* sp. can increase germination and seedling growth (Muñoz-Rojas et al. 2018).

In the same context, the use of cyanobacteria helps in crop protection against the adverse effects of herbicides. *Arthrospira platensis* suspension applied to faba bean seeds was found to improve the harmful effects of Fusillade herbicide and caused an increase in root and shoot protein and amino acid content. Moreover, the same treatment increased the antioxidant enzymes and reduced the lipid peroxidation and proline content of the faba bean plants (Osman et al. 2016).

Furthermore, Abo-Shady et al. (2018) found that priming barley grains in the cyanobacterial suspension of *Nostoc muscorum* before cultivation removes the toxic effect of granstar herbicide on yield parameters (number of spikes/plant, spike length, the weight of spike, number of grains/spike and weight of 100 grains) of barley plants. Besides, it stimulated the production of some amino acids which were not present in the control plant and increased the concentration of the amino acids that were previously present.

## The Mechanistic Approach Behind the Improvement and Protection of Plants by Using Cyanobacteria

Once cyanobacteria are exposed to oxidative stress, various compounds, such as antioxidants and secondary metabolites, can accumulate in cyanobacterial cells as an adjusted response to stress conditions (Kosar et al. 2015). Some enzymatic antioxidants such as superoxide dismutase (SOD), catalase, and glutathione peroxidase are created in microalgal cells due to abiotic stresses to defend against ROS-produced oxidative agents (Pikula et al. 2019). Likewise, cyanobacteria biostimulants inoculation in soil or foliar application has been shown to support the antioxidant activity of treated plants, hence alleviating the effects of stress-induced free radicals by direct scavenging and avoiding ROS formation (Ertani et al. 2019).

Cyanobacteria are a vital source of many biologically active compounds that can develop agricultural productivity. Cyanobacteria, use as crude or pure extracts. Furthermore, this range of metabolites influences crops’ production and improvement in many ways: (a) some extracts prompt higher crops’ productivity via an improvement in the soil quality (Table 4); (b) some metabolites perform straight on plant growth motivation (Table 4); and (c) others enrich crops’ progress through induce the protection against biotic and abiotic stress (Table 7). Additionally, cyanobacteria play an important role in the biogeochemical cycles of nitrogen, carbon, and oxygen, which is a feature of great significance in agricultural systems (Gonçalves, 2021).

**Table 7:** Cyanobacteria-induced plants protection against different abiotic stresses

Crop plants and stress	Cyanobacteria	Improvement effect	References
Salinity Mentha piperita fields	*Anabaena vaginicola* ISB42, *Anabaena oscillarioides* ISB46, *Anabaena torulosa*, *Anabaena sphaerica* ISB23, *Trichormus ellipsosporus,* and *Nostoc calcicola*	Stimulated germination percentage by 88, 87, 89, 86, 87, and 87%, respectively	(Shariatmadari et al. 2015)
Salinity Lettuce plants	*Oculatella lusitanica* LEGE	Increasing the non-enzymatic antioxidant reduced glutathione (66.67%) and decrease oxidative stress markers (H_2_O_2,_ MDA, and proline by 32.5%, 21.43%, and 12.5%, respectively)	(Brito et al. 2022)
High or low temperatures, salinity, and drought Poplar plants	Aphanothece *halophytica*	Induced heat shock protein-like 70 (HSP70) and Photosynthetic activity (1.4-fold)	(Takabe et al. 2008)
Irradiation, extreme temperatures, herbicides, oxidative, drought, and heat stress Tobacco plants	*Anabaena* sp.	Induction of protein pattern by 1.5-fold	(Gharechahi et al. 2015)
Fusillade herbicide Faba bean	*Arthrospira platensis*	Decreased root and shoot proline content by 42.9% and 33.3%, respectively. Also, reduced the lipid peroxidation (MDA content by 98.7%)	(Osman et al. 2016)
Granstar herbicide Barley plants	*Nostoc muscorum*	Increased yield parameters [number of spikes/plant (90%), spike length (23.5%), the weight of spike (1.13-fold), number of grains/spike (82.4%), and weight of 100 grains (92.3%)]	(Abo-Shady et al. 2018)
Brominal herbicide Wheat plants	*Arthrospira platensis*	Induced by 1.13-fold for chl a, 1.05-fold for chl b, 88.89% for carotenoids, and 90.63% for total pigments, and also by 41.49 and 58.62% for carbohydrates and protein content, respectively	(Osman et al. 2022)

In the same context, Gaafar et al. (2022) reported biochemical and molecular evidence explaining the protective effects of cyanobacteria, *Arthrospira platensis,* and *Nostoc muscorum*, for wheat crop plants against practice-specific herbicides. Priming wheat grains in the aqueous extract of these cyanobacteria stimulated the growth of the plants, especially after Broxyminyl herbicide spraying. Induction of antioxidant-defense enzymes, such as SOD, CAT, GPX, GST, and the non-enzymatic GSH molecules, was enhanced with a special performance for *Arthrospira platensis* treatment. Furthermore, detoxification genes, including GST (*GSTZ*, *GSTU*, and *GSTL*), *TaGS*, and *TaGPX*, were upregulated as a response to *Arthrospira platensis* and *Nostoc muscorum* application in combination with Brominal herbicide, which led to alleviating the toxic effect of Broxyminyl on wheat plants.

**Fig. 5:**
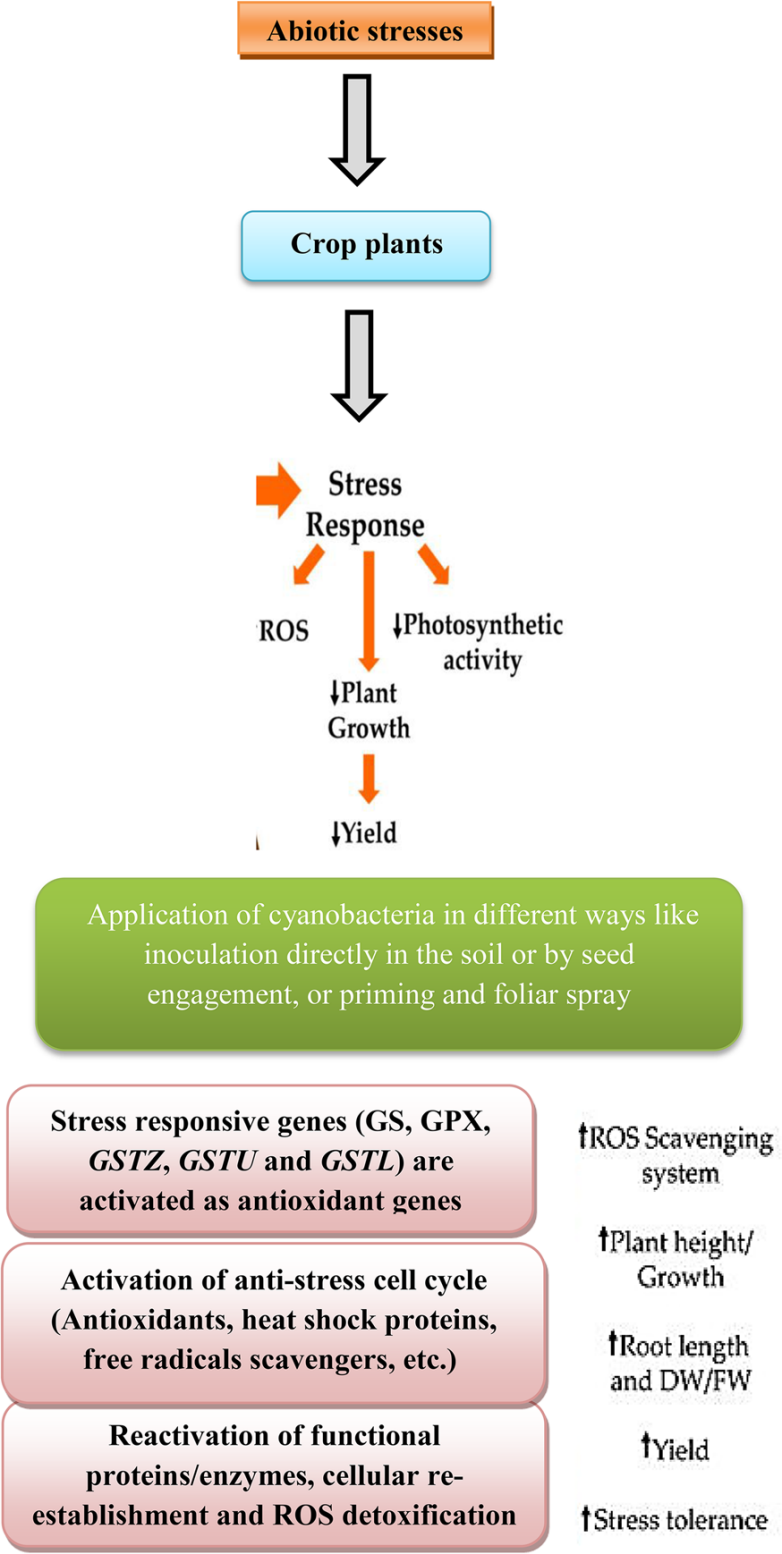
The mechanistic approach behind the improvement and protection of plants by using Cyanobacteria

The transformation of plants with genes consequent from cyanobacteria has been significantly established since the 1990s, with genes complicated in carbon metabolism, fatty acid biosynthesis, and pigment biosynthesis (Ahmad et al. 2016). Transformation with the gene like heat shock protein 70 (HSP70) *Aphanothece halophytica* rises its tolerance against high or low temperatures, drought, and salinity (Takabe et al. 2008). Likewise, the overexpression of genes of flavodoxins from Anabaena sp. upsurges tobacco plant tolerance against numerous stresses (drought, high irradiation, risky temperatures, herbicides, and water shortage (Gharechahi et al. 2015; Li et al. 2017). Also, oxidative stress decrease has been shown with the transformation of tobacco plants with the gene that codes for a ferredoxin-NADP+ reductase (Gir´o et al. 2011). Finally, Fig. 5 represented a suggested mechanistic flow diagram approach behind the improvement and protection of plants by using Cyanobacteria.

## Conclusion and future prospective

Plants exposed to risky environmental conditions cause abiotic stress, which can negatively impact crop production and development. To resolve this issue, the use of cyanobacteria can be a very hopeful option, as these compounds can (a) encourage the accumulation of antioxidant compounds, thus increasing the plant's tolerance to oxidative stress conditions; (b) improve the complete performance of higher plants, therefore stimulating their growth; (c) produce a wide variety of secondary metabolites with the ability to induce several plant protective mechanisms; and (d) chelate oxidizing numerous toxicants, xenobiotics, and complex organic compounds in the soils so that their bioremediation. Thus, they provide a lot of money spent on agriculture leading to nutrient-wealthy food that is healthy for the growth of the world population. Because of its safety and benefits, the mitigation strategies contesting unfavorable environmental conditions using cyanobacteria are important for stimulating the defensive mechanism system of plants against chemically hazardous pollutants and different abiotic stresses.
